# Targeting the Cancer–Neuronal Crosstalk in the Pancreatic Cancer Microenvironment

**DOI:** 10.3390/ijms241914989

**Published:** 2023-10-08

**Authors:** Ylenia Capodanno, Michael Hirth

**Affiliations:** 1 Institute of Pharmacology, Medical Faculty Heidelberg, Heidelberg University, Im Neuenheimer Feld 366, 69117 Heidelberg, Germany; 2 Department of Medicine II, Medical Faculty Mannheim, Heidelberg University, Theodor-Kutzer-Ufer 1–3, 68167 Mannheim, Germany

**Keywords:** pancreatic ductal adenocarcinoma, microenvironment, neuron, crosstalk

## Abstract

Pancreatic ductal adenocarcinoma (PDAC) represents one of the most aggressive solid tumors with a dismal prognosis and an increasing incidence. At the time of diagnosis, more than 85% of patients are in an unresectable stage. For these patients, chemotherapy can prolong survival by only a few months. Unfortunately, in recent decades, no groundbreaking therapies have emerged for PDAC, thus raising the question of how to identify novel therapeutic druggable targets to improve prognosis. Recently, the tumor microenvironment and especially its neural component has gained increasing interest in the pancreatic cancer field. A histological hallmark of PDAC is perineural invasion (PNI), whereby cancer cells invade surrounding nerves, providing an alternative route for metastatic spread. The extent of PNI has been positively correlated with early tumor recurrence and reduced overall survival. Multiple studies have shown that mechanisms involved in PNI are also involved in tumor spread and pain generation. Targeting these pathways has shown promising results in alleviating pain and reducing PNI in preclinical models. In this review, we will describe the mechanisms and future treatment strategies to target this mutually trophic interaction between cancer cells to open novel avenues for the treatment of patients diagnosed with PDAC.

## 1. Introduction

Pancreatic ductal adenocarcinoma (PDAC) is currently ranked as the fourth leading cause of cancer-related death worldwide, with a 5-year survival of less than 10% [1]. Despite the efforts made, there has been no major improvement in therapeutic progress against this disease in recent decades. To date, surgical resection is the only potentially curative treatment, but this is only eligible for patients diagnosed at an early stage, which accounts for only 11% of all cases [2,3]. For advanced-stage cancer cases, surgery, chemotherapy and radiotherapy are used to extend survival or relieve the patients’ symptoms. However, there is still no definite cure for most patients [1]. Most patients are asymptomatic at early stages, and symptoms that occur, such as abdominal pain and unexplained weight loss, are non-specific and thus remain unnoticed until later stages of the disease [3]. At the time of diagnosis, the disease is often advanced. The main symptom presented by up to 73% of patients is severe abdominal pain. In only 30% of these patients, abdominal pain is associated with pancreatic cancer in the early stage. [4]. Considered a negative prognostic factor for survival, severe pain not only greatly influences the quality of life for such patients but also may increase their risk of drug abuse [4].

Although mechanisms of pain generation in PDAC are only partially understood, it is widely accepted that pain sensation occurs due to prominent neuronal remodeling [4,5]. Neuronal remodeling is characterized by hypertrophied nerves and occurs as early as in the PanIN stage [5,6,7]. The underlying mechanism leading to neuronal remodeling is perineural invasion (PNI) of cancer cells. PNI is considered a hallmark feature in PDAC and it has been shown to correlate with poor prognosis, early cancer recurrence and cancer-associated pain [5,8,9]. One of the first hypotheses describing the occurrence of PNI revealed that cancer cells, as well as the nerve fibers, will choose the path of “least resistance” and move along this path to invade neighboring organs. In recent years, however, it has become evident that the cancer–neuronal interaction is not a passive process but a complex and highly specific interaction involving multiple signaling pathways [10]. Thus, the previously defined one-way “neurotropism” of cancer cells towards nerves is now described as a bidirectional crosstalk of signaling molecules produced by both the cancer cells and the nerves. In this scenario, neurons are able to control cancer initiation, growth and metastasis, whereas cancer cells induce functional alterations of the nervous system, including neuronal remodeling and neural inflammatory cell infiltration [7,11,12].

**Figure 1:**
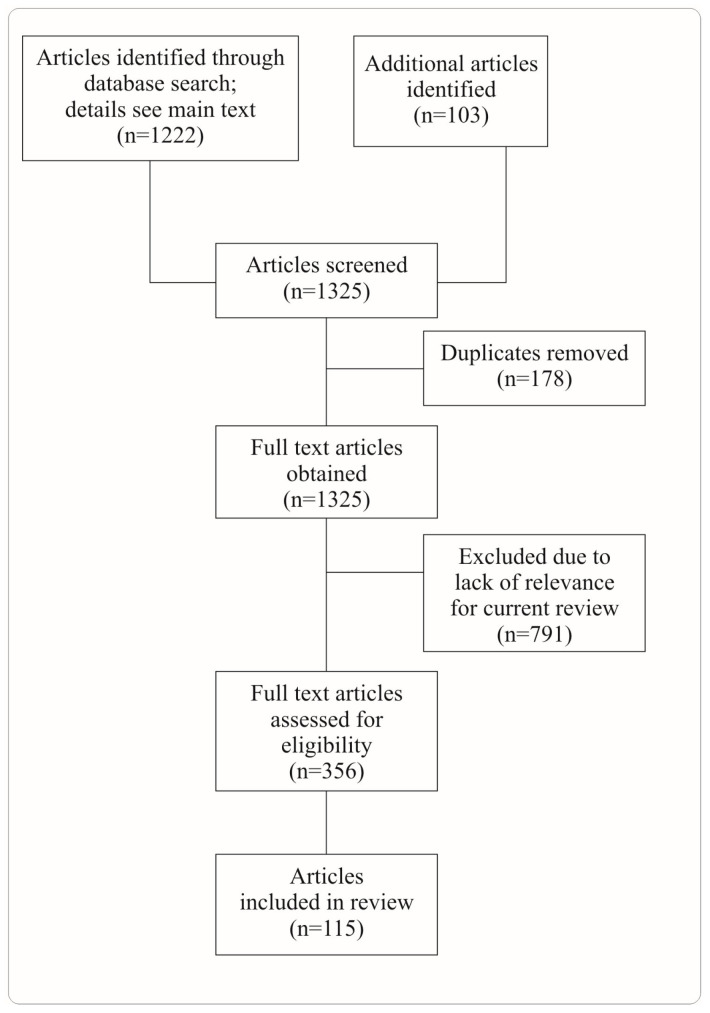
PRISMA flow diagram on article selection. We performed a literature search in PubMed using these terms: “(pancreatic cancer [title]) AND neuron”, “(pancreatic ductal adenocarcinoma [title]) AND neuron”, “(pancreatic cancer [title/abstract]) AND neuron [title/abstract]”, “(pancreatic cancer [title/abstract]) AND sympathetic [title/abstract]”, “(pancreatic cancer [title/abstract]) AND parasympathetic [title/abstract]”, “(pancreatic cancer [title/abstract]) AND ablation [title/abstract]”, “(pancreatic cancer [title/abstract]) AND neuronal [title/abstract]”, “(pancreatic cancer [title/abstract]) AND neural [title/abstract]”.

Recent evidence suggests that the crosstalk within nerves and cancer cells is due to the neurotrophic attributes of the tumor microenvironment (TME) [13,14,15]. In recent years, the impact of the nervous system as an integral part of the TME has gained increasing attention [11,13,14,15,16]. Considering that its stroma-rich TME limits the access of systemic therapies to cancer cells and contributes to poor clinical outcomes, understanding the composition of the TME and its role in PNI will represent a major milestone in the development of new therapeutic strategies for PDAC. In this review, we aim to elucidate which mediators are involved in cancer–neuronal interaction and have an impact on changes in the neuronal architecture and tumor biology (Figure 1). In particular, we highlight which of these mediators are potential targets to investigate in current (and future) clinical trials.

## 2. Clinical Relevance of Cancer–Neuronal Crosstalk

**Figure 2:**
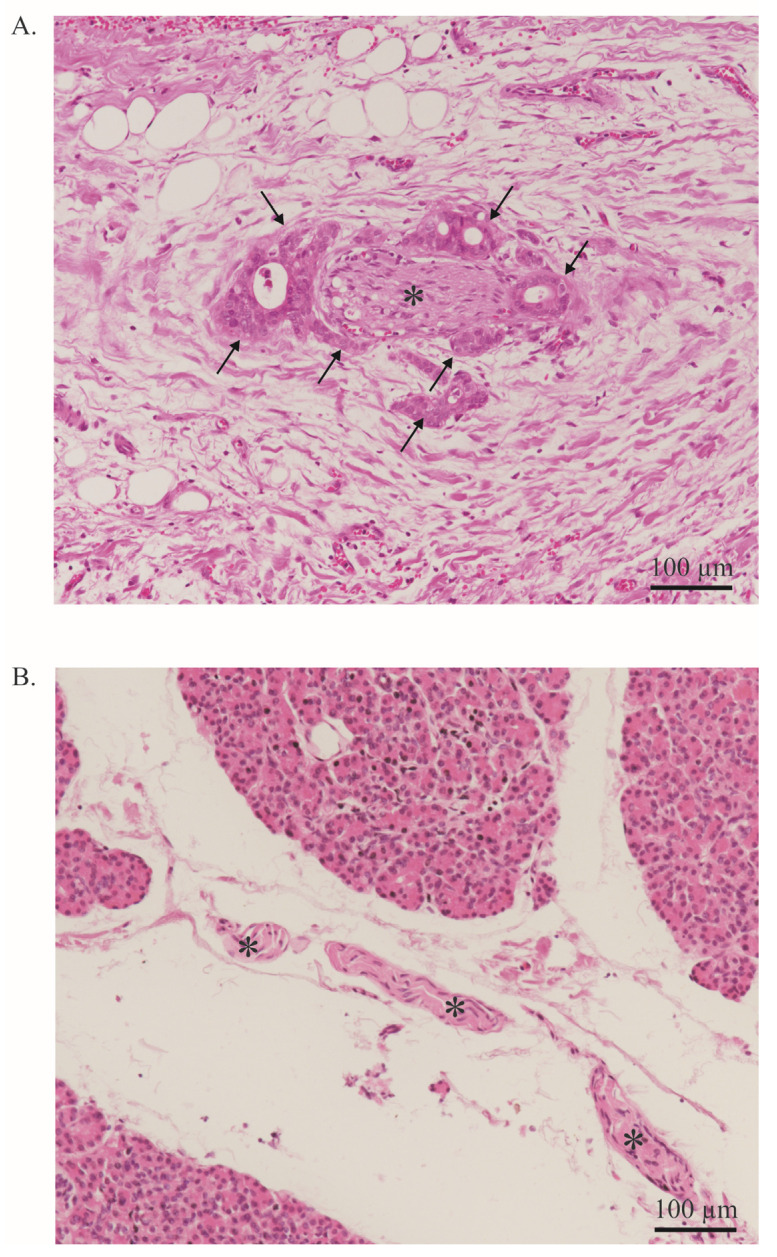
Neural invasion. (**A**). PDAC cells (arrow) infiltrate a nerve (asterisk). More than 33% of the circumference of the nerve is affected (perineural invasion). Stained with Hematoxylin and Eosin. Image at 20× magnification. (**B**). Healthy pancreas with a nerve bundle (asterisk). Stained with Hematoxylin and Eosin. The entire tissue shown represents the exocrine part of the pancreas. Endocrine parts are not shown. Image at 20× magnification.

Besides its aggressive behavior and poor response to treatment, another major feature of PDAC is PNI, which is defined as cancer cells surrounding at least 33% of the epineurial, perineural, and endoneurial space of the nerve sheath. PNI is present in virtually all patients [10,17]. This is a significant difference from other solid tumors in which PNI is less common [18]. PNI can be quantified using scoring systems and is primarily based on its extent and frequency (Figure 2). Interestingly, in PDAC, a significant positive correlation was found between the extent of PNI and patient survival [18,19,20]. PNI is an independent risk factor for the development of R1 resection and tumor recurrence [10,19] and an important predictor of metastatic spread along the neuronal compartment [8,17]. For instance, in a retrospective study, Takahashi et al. showed how micrometastases can be frequently found in the neuronal compartment of healthy pancreatic sections of PDAC-bearing patients [17]. This event, termed ‘intrapancreatic extratumoral perineural invasion (NEX) phenomenon’, was found in more than 50% of patients undergoing curative surgery. The occurrence of the NEX phenomenon was positively correlated with NI, whilst overall survival was negatively correlated with NEX, as NEX+ patients had a significantly worse survival compared to NEX- (350 vs. 1042 days). All patients who survived PDAC without tumor recurrence were NEX- [17].

In addition, PNI also has an impact on neuronal architecture. Specifically, extensive nerve fiber hypertrophy and elongation or sprouting of nerve fibers have been usually correlated with PNI occurrence [21,22]. Nerve fiber hypertrophy appears to be a major contributor to the development of cancer-associated pain in PDAC. Approximately 80% of patients develop cancer-associated pain during the progression of the disease [21,22]. Even with modern analgesic therapies, cancer-associated pain cannot always be controlled, thus prompting the quest for novel therapeutic targets. Interestingly, two retrospective studies showed that neoadjuvant therapy significantly reduced the rate of PNI [23,24] As the role of neoadjuvant therapy is currently not well established, this is an interesting observation [25]. For instance, Barbier et al. showed that neoadjuvant chemoradiation significantly reduced the rate of PNI from 93 to 43% [23]. However, neoadjuvant chemoradiation also prevented more than half of patients from receiving resection due to cancer progression. Consequently, it is unsurprising that neoadjuvant chemoradiation did not improve overall survival compared to direct resection [25]. Whether neoadjuvant therapy has a positive impact on patient survival or on the development of cancer-associated pain, e.g., as part of an extended multimodality therapy concept, remains to be elucidated.

## 3. Effect of Cancer—Neuronal Crosstalk on PDAC-Associated Pain

In addition to the poor prognosis, cancer-associated pain is a major problem in the clinical care of patients [26]. Cancer-associated pain occurs frequently and becomes progressive with increasing tumor development. Eventually, about 40% of PDAC patients describe it as severe [26]. The mechanism behind the development of this cancer-associated pain is still largely unclear. In recent years, however, more attention has been paid to the topic, so we will explore some of the most recent theories below.

An important observation was made by Ceyhan et al. in a landmark study where specimens of 546 patients with malignant and benign lesions of the pancreas and chronic pancreatitis were investigated [21]. Here, it was shown that the architecture of the nerves in PDAC, but not in benign lesions of the pancreas, undergoes significant changes. These changes, termed neuronal remodeling, mainly involve hypertrophy and proliferation of nerve fibers. Nerve fiber hypertrophy was also observed in the pancreatic parenchyma surrounding the PDAC lesion. In contrast, nerve fiber density exhibited a tendency to decrease toward the center of the tumor in PDAC [21]. Furthermore, Ceyhan et al. observed that patients suffering from severe cancer-associated pain also exhibited pronounced PNI. The extent of PNI correlated with neuronal remodeling, i.e., nerve fiber hypertrophy and increased nerve fiber density. Neuronal remodeling correlated with the extent of neuritis. Although these observations are descriptive, it appears evident that neural remodeling is influenced by PNI [21].

Few studies addressed neuronal remodeling and cancer-associated pain in animal models [27,28]. As the tumor cells invade the neuronal compartment, neuronal remodeling develops. These changes were found to be particularly prevalent in areas of high stromal activity, strengthening the idea of neurons as a critical microenvironmental element in PDAC [28]. Such changes were correlated with a significant overexpression of several chemokines, e.g., CX2CL1 and CXCL16, which are known to play a role in the development of neuropathic pain and cancer–neuronal crosstalk [28]. In addition, high NGF concentrations are also associated with increased nerve fiber hypertrophy and cancer-associated pain [26,29]. If these observations are correct, it should be possible to achieve an analgesic effect by inhibiting PNI. In our previous translational study, we showed that various chemokines play a central role in bidirectional cancer–neuronal crosstalk [30]. Among them, the chemokine axes CCL21-CCR7 and CXCL10-CXCR3 turned out to be the most promising. The chemokines were secreted by neurons and led to the attraction and targeted migration of PDAC cells carrying the corresponding receptors. In an orthotopic PDAC animal model known to develop cancer-associated pain, administration of CXCL10- or CCL21-neutralizing antibodies significantly reduced the extent of nerve fiber hypertrophy and, consequently, the development of cancer-associated pain. In human specimens, we demonstrated that patients suffering from preoperative cancer-associated pain had higher expression of the receptors CCR7 and CXCR3, respectively, than patients without cancer-associated pain. Thus, inhibition of these chemokine axes could represent a completely novel therapeutic approach, potentially preventing cancer-associated pain [30]. Further studies showed that intrathecal administration of a CX3CR1 antagonist also reduces cancer-associated pain [31]. However, it may be assumed that inhibition of further mediators of cancer–neuronal crosstalk could potentially also inhibit PNI, nerve fiber hypertrophy and cancer-associated pain. Unfortunately, little has been studied on the topic so far. Currently used opiate and non-opiate analgesics primarily cause neurolysis of the coeliac plexus in cases of severe cancer-associated pain. Still, benefits to patients’ quality of life are highly variable [32,33,34]. In the future, the use of inhibitors against these chemokines might be considered as a preventive strategy to reduce cancer-associated pain at the time of diagnosis.

Besides neural remodeling, other mechanisms of pain development in PDAC have also been investigated. It has been hypothesized that mediators released by tumor cells lead to neuronal sensitization. The best example is NGF, which physiologically leads to peripheral and central sensitization of nerve fibers in adults [35]. NGF thereby binds to trkA on neurons and the complex is endocytosed and retrogradely transported to the DRG, leading to changes in gene transcription. These include upregulation of NaV1.8, TRPV1, voltage-gated calcium channels, ASIC, substance P, CGRP, BDNF and others [36].

Current studies have not yet reflected the whole complexity of these mechanisms in the TME. In particular, it should not be forgotten that not only neurons are involved in the generation of pain. Demir et al. showed that Schwann cells are activated by hypoxia or PDAC-derived IL6 [37]. Remarkably, the authors showed that CXCR4- and CXCR7-expressing Schwann cells are chemoattracted to pancreatic cancer cells. Through a CXCL12-dependent-mechanism, activated Schwann cells suppress spinal astroglia and microglia activity, which is associated with less abdominal pain in vivo. This mechanism may allow the tumor to remain indolent in the early phase, making early diagnosis challenging. Here, the authors suggest that peripheral and central CXCL12-mediated signaling exert contrasting effects on nociception. The following represents another example of the exploitation of the microenvironment by tumor cells and specifically glial cells [37].

Furthermore, the interaction in the TME is most likely even more complex. For example, in glioblastoma, several studies demonstrated that microglia and macrophages interact with glioma stem cells, resulting in establishing an immunosuppressive microenvironment, promoting drug resistance and enhanced tumor progression [38].

## 4. Effect of Cancer–Neuronal Crosstalk on Cancer Growth and Invasiveness

Multiple studies have analyzed the cancer–neuronal crosstalk in vitro and in vivo [39,40]. In a genetic mouse model (LSL-Kras+/G12D; LSL-Trp53+/R172H; Pdx1-Cre; KPC), cancer–neuronal crosstalk was shown to occur very early in malignant transformation. Already in the PanIN stage, a significant increase in pancreatic innervation is evident, influencing tumor growth [39]. In vitro, when tumor cells and DRG neurons are co-cultured in a migration chamber, tumor cell outgrowth is directed to the neurons [40]. Vice versa, neurite outgrowth from cultured DRG neurons is directed to cancer cells, allowing them to migrate retrogradely along the neurite outgrowth [41,42].

**Figure 3:**
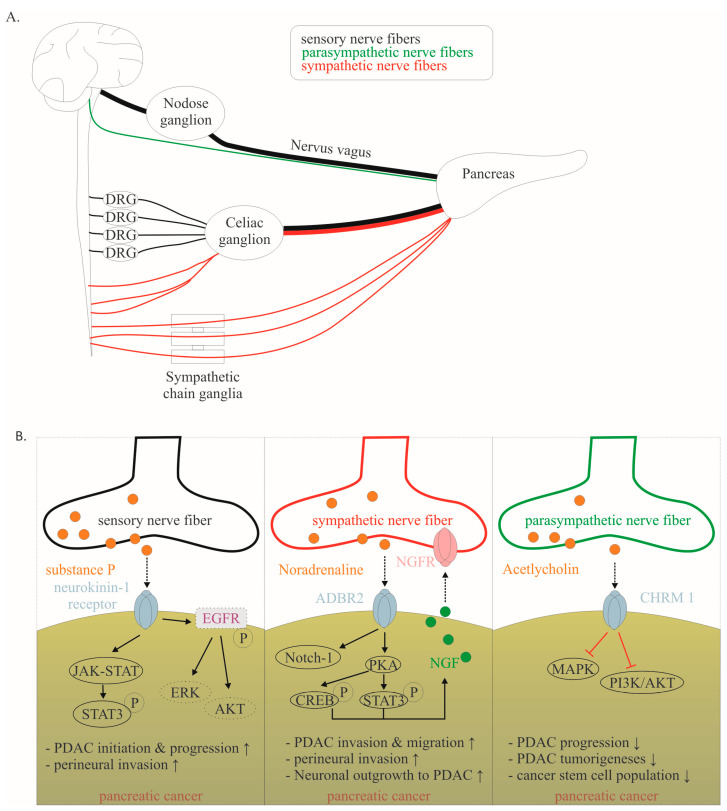
(**A**). Schematic illustration showing the routes of sensory, sympathetic and parasympathetic nerve fibers. (**B**). Mechanisms of cancer–neuronal crosstalk are shown separately for sensory, sympathetic, and parasympathetic neurons. Solid, black lines with an arrow symbolize activation, whereas red lines with a bar symbolize inhibition. Some signaling mechanisms are described in non-PDAC cancers. These are labeled with a dotted line. Their role in PDAC has to be confirmed in the future. The effect of interaction (↑ increase; ↓ decrease) is listed below for each mediator. Data collected from [12,16,40,42,44,53]. Abbreviations: β-adrenergic receptor 2 (ADBR2), nerve growth factor (NGF).

To understand the effects of this crosstalk on PDAC progression, it is important to highlight that the innervation of the pancreas is complex, including sympathetic, parasympathetic, and sensory nerves (Figure 3A). The intrapancreatic sympathetic nerves originate from the lower thoracic and upper lumbar segments of the spinal cord. Finally, sympathetic fibers reach the paravertebral ganglia of the sympathetic chain or the celiac ganglia and are evenly distributed in the pancreatic parenchyma (Figure 3A) [43]. The parasympathetic nerves primarily originate from the dorsal motor nucleus of the nervus vagus, of which up to 80% of the nerve fibers are sensory (Figure 3A) [12,16]. Further sensory input arises from sensory nerve fibers from caudal DRGs (TH8-T13) [43]. The sensory innervation of the pancreas is densest in the pancreatic head and contains approximately equal input from the vagus nerve and coeliac plexus [43]. In vivo studies have shown a remarkable switch in the pancreatic innervation quality during tumorigenesis. A significant increase in the sensory innervation of the pancreas can be found in KRASG12D-driven PDAC mouse models [8]. In both murine and human PDAC models, sensory nerves promoted the proliferation of PDAC cells via substance P/Neurokinin 1 receptor signaling and JAK–STAT pathway activation (Figure 3B) [16]. Neurokinin 1 receptor is overexpressed in PDAC cells [44]. Substance P promotes neurite outgrowth and attracts PDAC cells [44]. Several groups independently demonstrated that neonatal chemical ablation of sensory nerve fibers delays the development of pancreatic intraepithelial neoplasia (PanIN) and the development of PDAC [45,46]. At the molecular level, the JAK-STAT pathway is likely to be the main pathway involved [6]. Recently, investigations in non-small-cell lung cancer revealed that Neurokinin 1 receptor is critically involved in cancer cell proliferation and migration. Neurokinin 1 receptor can transactivate epidermal growth factor receptor (EGFR) phosphorylation, resulting in intracellular signaling via ERK and AKT [47]. However, in PDAC, the following mechanism remains to be elucidated. Accordingly, sensory ablation significantly prolonged survival in genetically engineered mouse models [45].

The sympathetic nervous system secretes catecholamines, which act on target organs via the α- and β-receptors [48]. It is known from epidemiological studies that chronic stress, which leads to increased catecholamine production, is associated with faster tumor growth. Accordingly, an increased concentration of norepinephrine is found in PDAC tissue [21]. In a landmark study, Renz et al. demonstrated the importance of catecholamines in PDAC [40]. Chronic neurophysical stress increases circulating epinephrine levels. The beta2-adrenergic receptor (ADBR2) is increasingly expressed in cancer tissue in the presence of a Kras mutation. The increased epinephrine levels lead to earlier tumor development and decreased survival by binding to ADBR2. Thereby, binding to ADBR2 leads to an increase in tumor growth and overexpression of NGF. This results in the attraction of additional nerves. Administration of an ADBR2 inhibitor antagonized this effect. In vivo, administration of an ADBR2 inhibitor also prevented cancer–neuronal crosstalk. This resulted in a reduction of neural outgrowth, reduction of tumor growth and a reduction of metastasis. In vivo, administration of an ADBR2 inhibitor or ablation of sympathetic nerve fibers resulted in a significantly improved response to chemotherapy [40]. In addition, in a large surgical cohort of 631 patients, the authors demonstrated that patients taking a nonselective beta-blocker (which also inhibits ADRB2) had a survival almost twice as long as those taking an ADBR1 inhibitor or not taking a beta-blocker. The outcome of this study was essentially confirmed. For example, a large Swedish registry study also showed that taking a beta-blocker was a highly significant independent factor for longer survival in PDAC [49]. Nonetheless, this study showed no difference between taking an unselective beta-blocker to ADBR1- or ADBR2 inhibitors, contrary to an even larger case–control study in over 4000 PDAC patients and 16,000 controls which showed that there was indeed a survival benefit primarily from taking a non-selective beta-blocker. The survival benefit was particularly evident when beta-blockers were taken for more than 2 years [50]. In a detailed US-based epidemiological study, it was shown that the use of a beta-blocker per se did not result in a survival benefit. Only the continuous use of beta-blockers before and after diagnosis did confer a survival advantage [51]. Currently, the optimal timing and duration of beta-blockade in PDAC remains to be determined. Since the survival benefit of beta-blockade in vivo was seen in stressed but not in unstressed animals, it also needs to be clarified whether beta-blockade may significantly improve survival only in a subgroup of patients [52]. However, since PDAC patients suffer from one of the highest levels of psychological distress, most patients may benefit from it [53]. The first clinical trials on the administration of non-selective beta-blockers in PDAC have recently started (Table 1; Table 1 summarizes the current status on clinical trials targeting mediators involved in cancer–neuronal interaction). Currently, trials on beta-blockade in advanced-stage PDAC are also simultaneously being planned. Prophylactic administration of beta-blockers may also be successful, although this has not yet been investigated in prospective clinical trials.

**Table 1:** Selected clinical and preclinical trials targeting the cancer–neuronal crosstalk.

Drug	Target	Current Status	Effect	Reference
Anti-NGF antibody or siRNA	NGF	Mouse model	Reduced neural outgrowth, reduced cancer growth, reduced metastasis	[54,55]
Trk-Inhibitor	Trk (unselective) or trkA (selective)	Phase I–II	NA	NCT03556228, NCT05046847, NCT02097810, NCT02568267, NCT04879121
Plerixafor	CXCR4 antagonist	Phase I–II	NA	NCT03277209, NCT02179970, NCT04177810
miR-383	ROBO3	Mouse model	Reduced cancer growth and metastasis	[56]
Minnelide	Several effects, including Inhibition des NF-κB	Phase I–II	NA	NCT03117920, NCT05557851, NCT03129139, NCT04896073
Propranolol	Beta adrenergic receptor	Phase II	NA	NCT03838029, NCT05451043
Celiac ganglion ablation	Denervation	Phase II–III	Reduction in cancer-associated pain, but no significant prolonged survival	[57]
Splanchnicectomy	Denervation	Phase III	Reduction in cancer-associated pain, prolonged survival only in presence of cancer-associated pain before intervention	[58]
Bethanechol	Muscarinic receptor M1	Phase I–II	NA	NCT03572283, NCT05241249

Abbreviation: NA (not available; e.g., because the study is still ongoing or study results are not yet available).

Furthermore, in response to catecholamines, tumor cells produce brain-derived neurotrophic factor (BDNF) [59]. Catecholamines bind to the ADRB3 on tumor cells for this purpose. BDNF increased the innervation through the TrkB receptors on neurons. BDNF knockdown inhibited not only neurite outgrowth but also stress-induced tumor growth [59]. However, BDNF has not yet been studied in detail, which is why clinical studies are not yet underway.

Parasympathetic nerves also play complex roles in PDAC. Enhanced cholinergic signaling can directly inhibit the MAPK/EGFR and PI3K/AKT pathways through CHRM1 and indirectly reduce the CSC population, suppressing tumorigenesis and cancer stemness [12].

Besides neuronal-derived mediators, various studies have shown that growth factors secreted from a specific type of cancer-associated fibroblasts, the pancreatic stellate cells (PSCs), have a major influence on tumor growth supporting cancer–neuronal crosstalk. For instance, in vitro studies have shown that supernatants of human pancreatic stellate cells can induce axonal sprouting, increased neurite density and perikaryon hypertrophy of DRG neurons. Additionally, co-culture system of PDAC cells and PSCs have shown an enhanced interaction between cancer cells and axonal DRG through secretion of extracellular matrix glycoprotein tenascin C by PSCs, which may be positively related to PNI, tumor stage and tumor recurrence. PDAC cells could also release sonic hedgehog signaling molecules to activate hedgehog signaling pathways in PSCs, leading to cancer invasion and nerve dysfunction. These findings indicate that PSCs support the communication between cancer cells and neurons in the TME, influencing tumor growth and invasiveness [60].

## 5. Ablative Strategies in PDAC to Reduce Cancer-Associated Pain and Tumor Growth

Multiple ablative therapies have been evaluated for pain relief and delayed tumor progression in palliative settings in PDAC. For instance, the interventional ablation of nerve plexi mostly involving the celiac plexus is frequently used. Patients who underwent coeliac plexus ablation tended to have a higher 1-year survival rate than the control group (16% vs. 6%) [61]. However, these procedures have essentially failed to show a significant survival benefit [34,57,62]. Surgical procedures to remove the surrounding nerve plexi also failed to show a clear survival benefit [63,64]. Therefore, this should only be performed for selected patients [65,66]. Similarly, resection of the splanchnic area in pancreatic left resection tends to even lead to a higher tumor recurrence rate [66]. Novel surgical procedures have been currently tested. This includes minimally invasive strategies for distal pancreatectomy with en bloc celiac axis resection using the retroperitoneal–first laparoscopic approach. It remains to be elucidated whether these might improve survival [67].

The question arises as to how ablative procedures can be optimized in the future. Pharmacological ablation of individual nerve fiber classes may yield more promising results in terms of prolonged survival in the future. For instance, instead of injecting alcohol, 6-hydroxy-dopamine could be used for (temporary) chemical sympathectomy or capsaicin for (temporary) peripheral sensory denervation [68]. In vitro and in vivo studies show that catecholamines increase tumor growth via β-adrenergic signaling [69]. Ablation of sympathetic nerve fibers is associated with prolonged animal survival and improved response to chemotherapy in animal models [40]. Mechanistically, norepinephrine leads to inhibition of apoptosis and stimulation of tumor growth by binding to the β-adrenergic receptor. Multiple signaling pathways are likely involved, such as MAPK and Notch-1 pathways. Activation of STAT3 also leads to overexpression of NGF, which further promotes neurite outgrowth (Figure 3B) [69,70]. However, if the vagus nerve (which contains sensory and parasympathetic fibers) was dissected, PanIN lesions developed earlier and PDAC development was premature [71]. Accordingly, survival of these animals was reduced. Moreover, increased neuronal activity of the vagus nerve prolonged survival in an in vivo metastatic pancreatic cancer mouse model [72]. Interestingly, in PDAC patients with perineurial invasion, the cholinergic neuronal content was reduced or nearly diminished (Figure 3B). These observations highlight that neurons exhibit pro- or anti-tumorigenic effects depending on the appropriate subtype. Therefore, it is unsurprising that ablation or resection of whole-nerve plexi is unlikely to provide a significant overall survival benefit.

However, a subgroup of PDAC patients may also benefit from procedures using alcohol ablation. For example, Lillemoe et al. performed laparoscopy in all patients diagnosed with PDAC. All 139 nonresectable patients were included in the study and randomized 1:1 (alcohol ablation in the splanchnic area vs. saline injection). Although an analgesic effect of splanchnicectomy was observed, there was no overall benefit on patient survival. Interestingly, splanchnicectomy resulted in a highly significant prolongation of survival only in patients who already experienced preoperative cancer-associated pain [58].

In conclusion, instead of ablating pro-tumorigenic contributing nerve fibers, another opportunity is activating anti-tumorigenic contributing nerve fibers. Activation of the parasympathetic nervous system is particularly suitable for this purpose. Under physiological conditions, parasympathetic innervation of the pancreas is important for organ development [73]. It stimulates the healthy exocrine pancreas and ablation of the vagus nerve leads to decreased pancreatic acinar growth [74]. Clinical studies suggest that increased vagus nerve activity slows the progression of PDAC development [72]. Accordingly, a higher rate of PDAC was shown in patients who received vagotomy for gastric ulcer disease in the past [72]. Furthermore, Renz et al. demonstrated in LSL-Kras+/G12D; Pdx1-Cre (KC) mice that vagotomy accelerated the development of PDAC, whereas treatment with the systemic muscarinic agonist bethanechol restored the normal KC phenotype [12]. In KPC mice with established PDAC, bethanechol improved the response to chemotherapy and significantly extended survival. These effects were mediated by the muscarinic receptor M1, which reduces the activity of the MAPK/EGFR and PI3K/AKT pathways [12]. It should be mentioned that acetylcholine, the neurotransmitter of parasympathetic nerve fibers, binds to both the muscarinic and nicotinic receptors. Binding to the nicotinic receptor promotes an immunosuppressive TME [75]. Thus, the selective activation of muscarinic acetylcholine receptor 1 with bethanechol represents a promising target. Accordingly, two clinical trials on the use of bethanechol in the preoperative setting and in combination with chemotherapy have recently started (Table 1). High binding specificity appears to be important in this regard, as, for example, overexpression of muscarinic acetylcholine receptor 3 is associated with increased lymph node metastasis and poorer overall survival [76]. Overall, it has been demonstrated that ablative procedures currently do not lead to a survival benefit. However, in the future, a selective approach on selected patient groups or more selective modification of distinct nerve fibers may contribute to a survival benefit. In particular, the recently developed virus-vector-based genetic local neuroengineering technology is a promising approach which is able to selectively manipulate specific types of nerve fibers innervating the TME [77].

## 6. Molecular Mechanisms of Cancer–Neuronal Crosstalk and Targeted Therapies

### 6.1. Neurons Are Attracted to PDAC Cells

**Figure 4:**
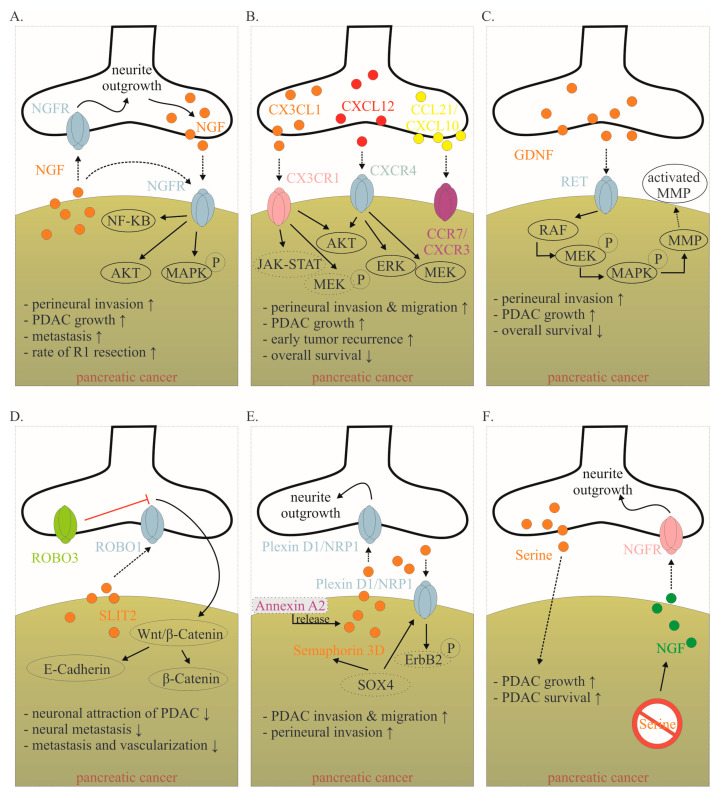
Mediators involved in the cancer–neuronal crosstalk. (**A**–**F**). Several mediators are involved in the close cancer–neuronal crosstalk, including NGF, chemokines (e.g., CX3CL1, CXCL10, CCL21), GDNF, SLIT2, Semaphorin 3D and Serine. Solid, black lines with an arrow symbolize activation, whereas red lines with a bar symbolize inhibition. Some signaling mechanisms are only described in non-PDAC cancers. These are labeled with a dotted line. Their role in PDAC has to be confirmed in the future. The effects of interaction (↑ increase; ↓ decrease) are listed below for each mediator. Data collected from [8,15,78,82,83,84,85,86,87,88,89]. Abbreviations: C-X3-C motif ligand 1 (CX3CL1); CX3C motif chemokine receptor 1 (CX3CR1); C-X-C motif chemokine 10 (CXCL10); C-X-C Motif Chemokine Receptor 3 (CXCR3); C-C Motif) Ligand 21 (CCL21); C-C chemokine receptor type 7 (CCR7); glial-cell-derived neurotrophic factor family of ligands (GDNF); nerve growth factor (NGF); nerve growth factor receptor (NGFR); Pancreatic ductal adenocarcinoma (PDAC); rearranged during transfection (RET); roundabout receptors (Robo); Slit glycoproteins (Slit). Red symbol crossing out Serine means Serine deprivation.

Many mediators are involved in this close cancer–neuronal bidirectional crosstalk. However, our understanding of this complex interaction is still very preliminary. Currently, it is known that PDAC cells secrete mediators that promote nerve invasion into tumor tissue [15]. Cancer cells activate physiological mechanisms which in homeostasis are necessary to provide organ innervations and nerve regeneration. Among the identified mediators, neurotrophins, particularly nerve growth factor (NGF), have been best studied (Figure 4A) [15,78]. During embryonic development, tissues that need to be innervated secrete NGF. Along the NGF gradient, neurons eventually innervate the target organ. In a healthy pancreas, NGF is barely detectable. However, as early as the PanIN stage, the amount of NGF doubled in PDAC cells. In the PDAC stage, the amount of NGF in tumor cells can increase up to seven-fold. Overexpression and secretion of NGF attracts neurons leading to neurite outgrowth into tumor tissue. In human tissue, a clear correlation has been reported between NGF overexpression and an increased extent of NI. Thus, it is not surprising that NGF concentration in tumor tissue is associated with an increased metastasis rate and increased probability of R1 resection. Microdissection studies demonstrated that both PDAC cells and nerves produce NGF and both express the corresponding receptors, so that a reciprocal interaction may be established over the time course of tumor disease [78].

NGF binds to two receptors: the high-specificity trkA receptor and the low-specificity p75NTR. Upon binding of NGF to the high-specificity receptor trkA, activation of MEK and MAPK pathways occurs, which promotes proliferation and suppresses apoptosis [79]. Binding to the low-affinity p75NTR inhibits proliferation and induces apoptosis [79]. In this regard, patients with a high trkA receptor have significantly reduced survival. Patients with high p75NTR expression showed significantly prolonged survival. Interestingly, NGF receptor expression was shown to be one of the most important predictive parameters in PDAC in a multivariable model [35].

The crucial role of NGF in this bidirectional crosstalk in PDAC has also been demonstrated in preclinical models. For instance, in vitro, knockdown of NGF or its receptors trkA and p75NTR, respectively, could limit the proliferation and migration of PDAC cells (Figure 4A). Also, inhibition of the NGF—trkA axis resulted in decreased migration of Mia PaCa2 cells toward DRG neurons and decreased neurite outgrowth [10]. Similarly, Lei et al. tested the following hypothesis using a robust siRNA (gold nanocluster-associated delivery of siRNA of NGF; GNC-siRNA) for NGF knockdown, which was used both in vitro and in vivo [54]. Application of GNC-siRNA reduced proliferation of Panc1 cells and inhibited migration of these tumor cells in a migration chamber. When Panc1 cells were co-cultured with DRG neurons, neurite outgrowth was directed towards Panc1 cells. Once Panc1 cells were pretreated with GNC-siRNA, the extent of neurite outgrowth decreased compared with untreated Panc1 cells. GNC-siRNA was also examined in vivo. Three PDAC mouse models were used (subcutaneous model, orthotopic model and patient-derived xenograft). After NGF knockdown in tumor cells, the amount of neurite outgrowth into tumor tissue was significantly reduced and tumor growth was reduced by approximately 50%. Then, depending on the tumor model, reduction of the metastatic rate varied from a discrete reduction to the virtually complete absence of distant metastasis. Thus, inhibition of the NGF axis represents a promising therapeutic option [54]. Human anti-NGF antibodies are already available and used in clinical trials. As NGF also plays an important role in the development and maintenance of pain in chronic inflammation, NGF antibodies have so far been used mainly in clinical trials on painful osteoarthritis [80]. The side effects mainly observed include headache, paresthesias and hypoesthesias [80,81]. Compared to aggressive chemotherapies, these side effects are considered acceptable. To date, no clinical trial for use in pancreatic cancer has been initiated, but this may soon follow due to the promising preclinical data. Specifically, the biweekly application of anti-NGF antibodies had favorable effects in LSL-Kras+/G12D; LSL-Trp53+/R172H; Pdx1-Cre (KPC) animals [55]. The development of PDAC was suppressed, the rate of PNI was reduced by 40% and the macrometastases were not detectable (vs. 30% in sham-treated KPC animals). Inhibition of the TrkA receptor also appears to be effective, although human trk inhibitors can cross the blood–brain barrier and thus the side effects to be expected might be more critical [55]. Nevertheless, first clinical studies in solid tumors, including pancreatic cancer, have been initiated (Table 1).

NGF also activates multiple pathways within the tumor that increase cellular invasiveness such as nuclear factor kappa–light-chain-enhancer of activated B-cells (NF-κB) [82]. In vitro data demonstrate that inhibitors of the NF-κB pathway inhibit NGF-mediated PNI and neural outgrowth. In vivo, inhibition of the NF-κB pathway leads to a reduction in neurotrophin expression, nerve density and PNI [82]. Inhibition of the NF-κB pathway was achieved on the one hand by NF-κB modulation plasmids and on the other hand by Triptolide and its water-soluble prodrug Minnelide. Triptolide is a diterpenoid triepoxide, which is extracted from the Chinese herb *Tripterygium wilfordii*. Among the over 300 ingredients of *Tripterygium wilfordii*, Triptolide is the most important bioactive component. The effect of triptolide is manifold [90,91]. On the one hand, it induces apoptosis in pancreatic cancer cells and elicits its antitumor activity through super-enhancer disruption to re-program cellular crosstalk [85]. On the other hand, it increases the cytotoxicity of various chemotherapeutic agents and inhibits the NF-κB pathway [82,91,92]. Therefore, it remains to be confirmed whether inhibition of the NF-κB pathway by triptolide leads to inhibition of cancer–neuronal crosstalk or whether other triptolide-induced pathways are responsible for the observed effects. Nevertheless, preliminary promising results on triptolide in PDAC are already available. For instance, in an orthotopic PDAC model, it was shown that the combined administration of Minnelide and paclitaxel significantly reduced tumor growth and improved survival [93]. In this model, the life expectancy in untreated animals was 13 days. After administration of paclitaxel or Minnelide as monotherapy, survival was significantly improved up to 21 days. By combined administration of Minnelide and paclitaxel, all animals were still alive after more than 6 weeks. Overall, compared to untreated animals, combined therapy reduced the rate of distant metastases by up to 90%. Based on these data, several clinical trials in PDAC (and other tumor entities) have recently been initiated (Table 1) [94].

### 6.2. PDAC Cells Are Attracted to Neurons

Chemokines are signaling molecules that exert the attraction and directional movement of leukocytes, as well as other cell types, including endothelial and epithelial cells. They are central mediators in the migration of cells, making it not surprising that several chemokines are involved in cancer–neuronal crosstalk [16].

Fractalkine (CX3CL1) is a well-studied chemokine released by neurons (Figure 4B) [95]. In the central nervous system, CX3CL1 plays an important role in neuron–glia crosstalk. Several PDAC cell lines express the corresponding receptor CX3CR1, which enables them to demonstrate PNI [95] (Figure 4B). CX3CR1 is not detectable in healthy human pancreatic tissue, whereas it is highly expressed in human PDAC tissue. Thereby, CX3CR1 expression correlates with the extent of NI, as well as local and early tumor recurrence [95]. The downstream mechanisms of CX3CR1 activation in PDAC need to be identified. However, it has been shown in prostate and breast cancer that the activation of the receptor leads to subsequent activation of several pathways, including PI3K/AKT, Raf/MEK/ERK and JAK/STAT, among others [96]. E6011 is a humanized IgG2 monoclonal antibody against human fractalkine, which has been used in phase II trials in inflammatory diseases such as rheumatoid arthritis. Clinical studies in PDAC have not yet been performed [83].

CXCL12 is also released from neurons and binds to the receptor CXCR4 (and CXCR7) on PDAC cells (Figure 4B) [83]. The activation of the CXCL12/CXCR4 axis significantly increased PDAC cells’ PNI and promoted neurite outgrowth. The expression of CXCR4 correlated highly significantly with the presence of NI in human specimens [97]. Inhibition of this pathway in vivo inhibited tumor growth and invasion of the sciatic nerve [97]. In an orthotopic PDAC mouse model, CXCR4 inhibition (using plerixafor) resulted in a significantly improved response to gemcitabine chemotherapy [98]. In human specimens, high CXCR4 expression but not high CXCR7 expression was associated with reduced overall survival [99]. While some gastric and colorectal carcinoma cells can produce CXCL12 themselves, the quantitative largest source in PDAC is derived from cancer-associated fibroblasts (CAFs) [100]. Physiologically, pancreatic stellate cells produce CXCL12 after tissue damage. In PDAC, cancer cells encourage CAFs to produce CXCL12 by secreting TNF-alpha, TGF-beta and others [100]. CXCL12 binding enhances PDAC proliferation via Akt, ERK and MEK signaling [101]. CXCL12 binding also increases treatment resistance through increased expression of pro-survival proteins Bcl-2, BclxL and Notch1 and inactivation of BAD [100]. In addition to its influence on cancer–neuronal crosstalk and tumor growth, the CXCL12/CXCR4 axis is also important for immune cell migration and possibly plays a role in modulating the response to immunotherapy [100,102]. Plerixafor is a selective CXCR4 antagonist approved to mobilize hematopoietic stem cells into the peripheral blood for collection and autologous transplantation in patients with non-Hodgkin lymphoma and multiple myeloma. Therefore, it is not surprising that CXCL12-CXCR4-axis also plays an important role in immune cell migration [100,102]. Based on the following findings, preliminary clinical studies are planned or underway. This will show the impact of the CXCL12–CXCR4 axis on cancer–neuronal crosstalk, tumor growth and response to immunotherapy.

Another prominent group of proteins secreted by nerves is the glial-cell-derived neurotrophic factor family of ligands (GDNF). The GDNF family ligands include GDNF, neurturin, artemin and persephin. GDNF family members bind to glycosylphosphatidylinositol anchor-linked GDNF family receptor alpha 1 (GFRα1), which recruits rearranged during transfection (RET) receptor tyrosine kinase for dimerization [84]. GDNF is highly expressed in the peripheral and central nervous system and stimulates the development, survival and differentiation of neuronal cells [84]. RET is expressed on several PDAC cell lines [84]. Neurons from mice deficient in GDNF had a reduced ability to attract cancer cells [15]. It has been shown that PNI is dependent on neuronal GDNF secretion and GDNF coreceptors RET and GFRα1 expressed in human PDAC cells (Figure 4C) [84]. High RET expression in human PDAC was established as a negative prognostic parameter [8]. Interestingly, PNI was blocked by treatment with PYP1, a potent RET inhibitor, showing how targeting nerves could serve as a potential mechanism to decrease PDAC growth [15]. After GDNF stimulated the RET mitogen-activated protein kinase cascade, it facilitated matrix degradation by producing more matrix metallopeptidase 2 (MMP2), MMP9 and MMP14, thereby accelerating cell invasiveness [8]. Monoclonal antibody-GDNF fusion protein was tested in parkinsonian monkeys. Focal pancreatic acinar to ductular metaplasia with transition to pancreatic intraepithelial neoplasia 1B (PanIN-1B) lesions were detected in several animals [103]. Whether this was due to the specific antibody fusion protein or to GDNF inhibition itself remains to be elucidated. Recently, protein neuroligin 1 (NLGN1) was shown to promote cancer cell invasion and migration along nerves [104]. Functionally, NLGN1 was shown to exert its effect in cooperation with GDNF (NLGN1-GDNF cooperation). Therefore, NLGN1 inhibition is a promising target for clinical trials.

Slit glycoproteins (Slit) and their roundabout receptors (Robo) are guide molecules in neuronal development, axon guidance, glial migration and angiogenesis [105,106]. Especially in PDAC, this pathway seems to play an important role in cancer–neuronal crosstalk and metastasis (Figure 4D). Thus, the expression of SLIT2 is reduced in PDAC tissue [85]. SLIT2 binds to ROBO1, which is expressed on neurons. Overexpression of SLIT2 in deficient PDAC cells reduces attraction by DRG neurons and thus PDAC migration along outgrowing neurons is inhibited [85]. Interestingly, even though the migration of PDAC cells towards neurons was reduced, their general motility was not affected. In vivo, restored SLIT2 expression was shown to reduce metastasis and vascularization [85]. Nevertheless, these findings remain to be confirmed, as the opposite effect was also observed for SLIT2-ROBO1 [107]. Inhibition of the WNT/β-catenin pathway may thus be the main mechanism by which the Slit/Robo pathway inhibits pancreatic cancer growth. Specifically, inhibition of the WNT/β-catenin pathway by Slit2/Robo signaling enhances the formation of β-catenin and E-cadherin complexes, increasing tumor cell adhesion and inhibiting tumor invasion and migration, thereby improving patient prognosis [86]. Interestingly, high expression of ROBO3, a known inhibitor of ROBO1/2 signaling, was associated with shorter survival in a cohort of 142 PDAC patients undergoing pancreatectomy with curative intent [56]. ROBO3 increases with clinical grade of PDAC and promotes cancer cell growth and metastasis in vitro and in vivo [108]. Robo3 can activate the WNT/β-catenin pathway, thus promoting pancreatic cancer growth and invasion [86]. Thus, the Slit-Robo pathway represents a very interesting target in PDAC. For example, miR-383 was identified as a suppressor of ROBO3 [108]. Besides its role in cancer–neuronal crosstalk, the SLIT-ROBO pathway is involved in other mechanisms of tumor growth and metastasis [86].

Another axon guidance molecule is Semaphorin 3D, which was found to be overexpressed in tumorigenesis [87]. Migration and PNI of PDAC is increased via secretion of Semaphorin 3D by pancreatic cells and activation of Plexin D1 on neurons (Figure 4E). Knockdown of Semaphorin 3D and loss of neural Plexin D1 reduces neurite outgrowth and metastasis in vivo [87]. Furthermore, high mRNA expression of Semaphorin 3D and Plexin A1, another molecule central to semaphorin signaling, were both associated with poor patient survival [86]. Secretion of Semaphorin 3D is increased by AnnexinA2. Annexin A2 is a metastasis-associated protein in PDAC that has been shown to be essential for the metastatic growth in genetically engineered spontaneous pancreatic tumor-producing KPC mice. By comparing tumor cells of AnnexinA2 wild type vs. knock-out KPC mice, Semaphorin 3D and PlexinD1 were among the most differentially expressed genes. Thus, disrupting the AnxA2/Sema3D/PlexinD1 signaling appears to be a promising therapeutic strategy for further clinical trials [87]. The complex signaling mechanisms of Semaphorin 3D are only partially understood. Interestingly, in non-PDAC cancer cells, the prometastatic activity of Semaphorin 3E is mediated by transactivation of PlexinD1-associated Erb2b (Figure 4E) [88].

Considering the PDAC environment is characterized by poor nutrition supply, it is becoming evident that PDAC cells are dependent on neurons to receive external growth stimuli [109]. Specifically, neurons secrete mediators stimulating cancer growth and supplying oxygen and nutrients [16,110,111]. For instance, amino acids are essential for PDAC growth. Serine is a conditionally essential amino acid that represents the second most abundant amino acid found in human proteins and can be secreted by neurons [89]. Serine is necessary for several metabolic pathways, highlighting its role in terms of tumor growth and survival in cancers [112]. For instance, human PDAC cell lines are completely dependent on exogenous serine (Figure 4F). If neurons are prevented from producing serine, tumors grow approximately 40% smaller [109]. Interestingly, after serine deprivation, PDAC cells massively overexpress NGF in order to recruit more neurons to meet its nutritional needs [109].

## 7. Conclusions and Future Directions

One reason for the poor response of PDAC to current therapies is its characteristic TME, which is responsible for tumor cell growth in an immunosuppressive milieu, poor penetration of chemotherapeutic agents and sufficient growth stimuli [113]. In PDAC, the cancer–neuronal interaction is very pronounced and represents a promising target out of the TME. Several preclinical studies have shown that interrupting the tight cancer–neuronal crosstalk can lead to very promising results. In addition, this also offers the possibility of reducing cancer-associated pain in PDAC, significantly increasing the quality of life of these patients. Even though preliminary clinical studies on the inhibition of cancer–neuronal axis are underway, there are still many obstacles to developing novel therapies. For instance, the current lack of efficient in vitro and in vivo models reproducing the disease remains one of the main shortcomings in studying the cancer–neuronal interaction. Particularly, only a few well-characterized in vivo models are available to date for the study of cancer-associated pain [27,28]. Additionally, it is now clear that the previous assumption of unidirectional crosstalk between these two entities is far too simplistic. As numerous data suggest, cancer–TME interaction is based on multiple cell types, e.g., cancer–neuronal–immune crosstalk. For example, tumor-specific sympathetic denervation downregulated the expression of programmed death-1 (PD-1), PD-L1 and FOXP3, suppressing tumor progression [114]. This highlights that combination therapies targeting different cancer–TME interactions constitute a promising future direction [115]. Uncovering these mechanisms through collaborations between experts from different fields will be compelling to develop novel treatment options for PDAC patients.

## Abbreviations

β-adrenergic receptor (ADBR); brain-derived neurotrophic factor (BDNF); cancer-associated fibroblasts (CAF); glycosylphosphatidylinositol anchor-linked GDNF family receptor alpha 1 (GFRα1); LSL-Kras+/G12D; LSL-Trp53+/R172H;Pdx1-Cre (KPC); nerve growth factor (NGF); perineural invasion (PNI); Nuclear factor kappa–light-chain-enhancer of activated B-cells (NF-κB); Pancreatic ductal adenocarcinoma (PDAC); Pancreatic intraepithelial neoplasia (PanIN); rearranged during transfection (RET); roundabout receptors (Robo); Slit glycoproteins (Slit); tumor microenvironment (TME)
